# Sex hormones and brain pathology in autosomal dominant AD: preliminary findings from the Colombia‐Boston (COLBOS) biomarker study

**DOI:** 10.1002/alz.095137

**Published:** 2025-01-09

**Authors:** Clara Vila‐Castelar, Averi Giudicessi, Ana Y Baena, David Fernando Aguillon, Francisco Piedrahita, Diana Munera, Liliana A Ramirez Gomez, Edmarie Guzman‐Velez, Vincent Malotaux, Jairo E. Martinez, Alex L Badillo‐Cabrera, Nikole A Bonillas Félix, Lusiana Martinez, Hang Lee, Karen K Miller, Jill M Goldstein, Francisco Lopera, Yakeel T. Quiroz

**Affiliations:** ^1^ Massachusetts General Hospital, Harvard Medical School, Boston, MA USA; ^2^ Grupo de Neurociencias de Antioquia, Universidad de Antioquia, Medellín Colombia; ^3^ Grupo de Neurociencias de Antioquia, Facultad de Medicina, Universidad de Antioquia, Medellín Colombia

## Abstract

**Background:**

Evidence suggests that a history of reduced estrogen exposure associates with greater Alzheimer’s disease (AD) risk. However, the association of sex hormone levels with AD biomarkers has not been studied. We examined plasma levels of sex hormones in males and females with autosomal dominant AD due to the E280A Presenilin‐1 mutation and age‐matched non‐carriers, and their relationship to AD brain pathology.

**Methods:**

Blood samples were collected from 55 females (mean age 38.9±8.5 years; 31 carriers) and 46 males (mean age 39.3±5.5 years; 23 carriers) from the Colombia‐Boston (COLBOS) study. Samples were collected before 10 am for males and postmenopausal females and within 7 days of menses onset (follicular phase) in premenopausal females. We measured plasma concentrations of estradiol and progesterone in females, and total testosterone in both males and females using liquid chromatography‐tandem mass spectrometry. Free testosterone was calculated from the laws of mass action using total testosterone and sex hormone binding globulin (SHBG) levels. A subset of 78 participants completed florbetapir‐ and flortaucipir‐PET. We conducted t‐tests and linear regressions.

**Results:**

Estradiol, progesterone and free testosterone levels did not differ between female carriers and non‐carriers (estradiol: carriers = 49±59pg/mL, non‐carriers = 71±75pg/mL, *p* = .25; progesterone: carriers = .44±.71ng/mL, non‐carriers = .32±.37ng/mL, *p* = .44; free testosterone: carriers = .3±.1ng/dL, non‐carriers = .2±.2ng/m:, *p* = .64). Testosterone levels did not differ between male carriers and non‐carriers (free testosterone: carriers = 10.0±3.2ng/dL, non‐carriers = 11.4±3.7ng/dL, *p* = .47; total testosterone: carriers = 468±126ng/dL, non‐carriers = 485±196ng/dL, *p* = .76). As premenopausal females age, female carriers showed lower estradiol levels than non‐carriers (*p* = .001); there were no differences in progesterone (*p* = .18) or free testosterone levels (*p* = .78). As males age, free and total testosterone levels did not vary between male carriers and non‐carriers (free testosterone: *p* = .99; total testosterone: *p* = .59). Among females, estradiol, progesterone, and free and total testosterone levels were not associated with amyloid burden or precuneus tau (*ps*>.05). Among males, free and total testosterone levels were not associated with amyloid burden or precuneus tau (*ps*>.05).

**Conclusions:**

Preliminary findings suggest that females with autosomal dominant AD may have lower estradiol levels as they age, while sex hormones were not related to amyloid or tau burden. Further research is needed to understand the effect of sex hormones on AD progression among males and females.

